# Could GILZ Be the Answer to Glucocorticoid Toxicity in Lupus?

**DOI:** 10.3389/fimmu.2019.01684

**Published:** 2019-07-17

**Authors:** Jacqueline K. Flynn, Wendy Dankers, Eric F. Morand

**Affiliations:** School of Clinical Sciences at Monash Health, Monash University, Melbourne, VIC, Australia

**Keywords:** GILZ, glucorticoids, lupus (SLE), transcription factor, treatment, regulation

## Abstract

Glucocorticoids (GC) are used globally to treat autoimmune and inflammatory disorders. Their anti-inflammatory actions are mainly mediated via binding to the glucocorticoid receptor (GR), creating a GC/GR complex, which acts in both the cytoplasm and nucleus to regulate the transcription of a host of target genes. As a result, signaling pathways such as NF-κB and AP-1 are inhibited, and cell activation, differentiation and survival and cytokine and chemokine production are suppressed. However, the gene regulation by GC can also cause severe side effects in patients. Systemic lupus erythematosus (SLE or lupus) is a multisystem autoimmune disease, characterized by a poorly regulated immune response leading to chronic inflammation and dysfunction of multiple organs, for which GC is the major current therapy. Long-term GC use, however, can cause debilitating adverse consequences for patients including diabetes, cardiovascular disease and osteoporosis and contributes to irreversible organ damage. To date, there is no alternative treatment which can replicate the rapid effects of GC across multiple immune cell functions, effecting disease control during disease flares. Research efforts have focused on finding alternatives to GC, which display similar immunoregulatory actions, without the devastating adverse metabolic effects. One potential candidate is the glucocorticoid-induced leucine zipper (GILZ). GILZ is induced by low concentrations of GC and is shown to mimic the action of GC in several inflammatory processes, reducing immunity and inflammation in *in vitro* and *in vivo* studies. Additionally, GILZ has, similar to the GC-GR complex, the ability to bind to both NF-κB and AP-1 as well as DNA directly, to regulate immune cell function, while potentially lacking the GC-related side effects. Importantly, in SLE patients GILZ is under-expressed and correlates negatively with disease activity, suggesting an important regulatory role of GILZ in SLE. Here we provide an overview of the actions and use of GC in lupus, and discuss whether the regulatory mechanisms of GILZ could lead to the development of a novel therapeutic for lupus. Increased understanding of the mechanisms of action of GILZ, and its ability to regulate immune events leading to lupus disease activity has important clinical implications for the development of safer anti-inflammatory therapies.

## Systemic Lupus Erythematosus (SLE)—A Diverse Chronic Autoimmune Disease

Systemic lupus erythematosus (SLE) is an incurable chronic disease, affecting ~1 in 1,000 people world-wide (1, 2), resulting in a marked loss of life expectancy and quality of life. The prevalence of SLE is higher in women, particularly of child bearing age, and in African American, Asian and indigenous Australian populations (1). SLE is one of the top 10 causes of death in young adult women (3), but the current treatment consists mainly of glucocorticoids (GC) which cause severe adverse effects (4).

SLE is characterized by multisystemic inflammation occurring in periodic disease flares, which can affect multiple organs such as the kidneys, lungs, brain, heart, blood, and skin. The most common symptoms include rash, arthritis, and fatigue. The heterogeneity of the disease makes clinical diagnosis and measurement very challenging, resulting in the use of clinical criteria to categorize patients with autoimmune disease as having SLE. For example, the American College of Rheumatology (ACR) classification criteria, which comprise 11 disease features, identifies patients who have at least four features as SLE (5). The requirement for multiple features highlights the heterogeneity of the disease. The majority of SLE patients are positive for antinuclear antibodies (ANA) which target the nuclear components of cells. The most commonly detected specific autoantibodies are dsDNA, anti-Ro and anti-Sm antibodies which also can act as markers for kidney disease (6). Furthermore, the presence of autoantibodies especially to Ro, La, Sm, and RNP is strongly associated with detection of interferon (IFN)-induced gene expression in peripheral blood (7). This profile of IFN-induced genes is termed the IFN signature, and is found in approximately 95% of children and 50–70% of adults with SLE (8–10).

Although the initiating cause of SLE is unknown, it is thought to result from a failure in tolerance checkpoints in several components of the immune system. This includes the escape and proliferation of autoreactive B cells, the development of autoantibodies, and the formation of immune complexes which can initiate organ inflammation and/or directly damage cells (8, 11).

### Production of Autoantibodies and Immune Cell Dysfunction

One theory regarding the initiation of SLE is impaired clearance of apoptotic cells. Phagocytes from SLE patients are less effective in clearing apoptotic cells, and the uncleared apoptotic cells present apoptotic bodies and nucleic antigens to the extracellular space (12). These nucleic antigens, if internalized via Fc receptor binding of nucleic acid immune complexes, activate toll like receptors (TLRs). Particularly TLR 7 whose ligand is ssRNA [associated with the production of anti-Sm antibodies in SLE (13)], and TLR 9 whose ligand is unmethylated CpG-rich DNA. Downstream signaling resulting in the production of multiple cytokines, including interleukin (IL)-6, IL-1, and tumor necrosis factor (TNF)-α, which play a pivotal role in immune cell dysfunction and chronic inflammation (14).

Importantly, this pathway also leads to production of type I IFN (IFN-α and IFN-β), an important hallmark of SLE (15). Type I IFNs can also be induced via TLR-independent pathways such as retinoic acid-inducible gene 1 (RIG-I), melanoma differentiation-associated protein 5 (MDA-5) and cyclic GMP-AMP synthase (cGAS) receptors which activate innate immune cells through the detection of cytoplasmic nucleic acids (16). The main producers of type I IFN are plasmacytoid DCs (pDC). Despite a reduction in the number of circulating pDC in SLE patients, these cells accumulate at inflamed sites, particularly the skin and kidneys, and secrete large amounts of type I IFNs (17, 18).

One of the actions of IFN-α is to prime mature neutrophils and assist the formation of neutrophil extracellular traps (NETs) (19, 20). NETs are mesh-like structures composed of chromatin fibers and nuclear components, designed to trap and kill microbes (21). However, when inappropriately cleared, these NETs are also a source of auto-antigens. Thereby, they contribute to the development of auto-antibodies, particularly against dsDNA, and the immune complexes that cause organ damage in SLE (22). Finally, by exposing intracellular nucleic acid antigens, NETs activate pDCs and further exacerbate the production of type I IFN, creating a cycle of type I IFN production and NET formation in SLE patients (20).

Components of apoptotic cells can also be taken up by antigen-presenting cells and will activate T and B cells through the normal antigen presentation pathway. CD4^+^ T cells from SLE patients display a high expression of CD40 ligand (CD40L) compared to healthy donors, which also assists in activation and differentiation of B cells due to its role as co-stimulatory molecule (23). T follicular helper cells are expanded in SLE patients and promote the differentiation of autoantibody producing B cells (24). Additionally, Th17 cells, which promote inflammation, are increased in SLE, whilst T regulatory cells (Tregs) are suppressed (25). Furthermore, increased T cell numbers in SLE provides more T cell help for B cell differentiation, survival and proliferation (25), as does an elevated level of B cell activating factor (BAFF; also known as B Lymphocyte Stimulator, BLyS or TNF like ligand, *TNFSF13B*). Overexpression of BAFF is associated with increased survival of activated autoreactive B cells and a decrease in self-tolerance which leads to lupus-like autoimmune disease in mouse models (26). BAFF also assists in B cell survival during differentiation and is associated with SLE disease activity (14). B cells, which are activated by CD4^+^ T cells, contribute to disease both via antibody production and antigen presentation to T cells. B cells in SLE are hyperactive and contribute significantly to the production of autoantibodies, cytokines and augmented antigen presentation to T cells (25). Naïve B cells are reduced in number in the blood of SLE patients, whilst there is an increase in plasmablasts leading to an increase in antibody production. The cycle of excess antibody production perpetuates inflammation via immune complexes, as noted above.

Thus, SLE is a diverse autoimmune disease mediated by the disordered activation of multiple immune cells causing widespread chronic inflammation, resulting in multi-system morbidity and making management an enormous challenge. To combat the diverse nature of the systemic inflammation, the broad effects of GC on immune cell function has led to them being used widely to elicit broad suppression of autoimmunity and its inflammatory consequences.

### Glucocorticoid Treatment of SLE

GC have been used for decades for the treatment of inflammatory and autoimmune diseases such as rheumatoid arthritis and SLE (27, 28). GC have a substantial impact on the immune system via the ability to regulate 1,000 of genes. Gene regulation occurs via GC binding to the GC receptor (GR), which is expressed in almost every cell in the body. Thus, the effects of GC are not tissue- or organ-specific and treatment with GC has the ability to target inflammation in multiple parts of the immune system, and in multiple organs, simultaneously. This is valuable in a disease like SLE, in which cell types are implicated and multiple organs are affected. In SLE, dosing of GC varies according to the severity of inflammation and the nature of the organs affected (28). Unfortunately, adverse effects of GC, chiefly metabolic, are also dose-dependent, and are seen almost universally in patients with SLE treated with GC long term (28, 29).

To reduce reliance on GC, and hence dose-dependent adverse effects, patients with SLE are usually also treated with additional immunomodulatory or immunosuppressive drugs, such as anti-malarials, mycophenolate mofetil, or azathioprine. These drugs are also broad spectrum in their effects, and associated with significant adverse effects. The only targeted therapy approved for the treatment of SLE is belimumab, approved by the FDA for use in 2011. Belimumab is a neutralizing human monoclonal antibody to BAFF, which is elevated in SLE, suggesting that a BAFF inhibitor has the potential to control B cell dysfunction in SLE (14, 26). Belimumab has been approved for treatment of patients with active autoantibody positive SLE who have not responded to conventional therapies (30). Belimumab does not induce a rapid clinical benefit and though its use is associated with reduced GC dosing (31), GC remains the treatment for the majority of SLE patients. Additional targeted therapies directed against key inflammatory cytokines for SLE, such as IL-6, have been found to be ineffective in Phase II trials in SLE, despite their efficacy in rheumatoid arthritis (32). This suggests upstream targeting of inflammatory signaling pathways is important for development of a GC replacement therapy.

That GC are the mainstay treatment for SLE despite its use being plagued with severe side effects, such as increased cardiovascular disease, osteoporosis and diabetes (28, 29, 33), highlights the lack of a viable alternative. Thus, there is a critical need for the development of new therapeutics with similar potent immune actions but without the detrimental metabolic effects.

## The Mechanism of GC Action in the Treatment of SLE

The main mechanism via which GC act on the immune system is through binding to the glucocorticoid receptor (GR). The GR is encoded by the *NR3C1* gene and has two major forms, GRα and GRβ, which are alternative splicing isoforms from N3C1 (34). GRα is the form that resides in the cytoplasm and is dependent on GC binding for function. The GR contains an N-terminal regulatory domain, central DNA binding domain, hinge region and C-terminal ligand binding domain (35–37). The GRα is located in the cytoplasm and forms a complex with several proteins including heat shock protein 70 and 90 (38). Upon ligand binding, GRα is released from this complex and can interact with cytoplasmic signal transduction molecules or translocate to the nucleus.

**Figure 1:**
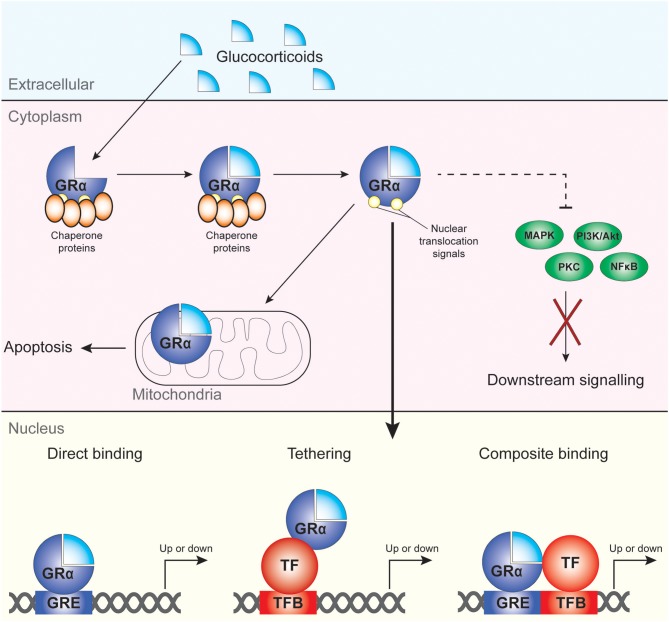
Mechanisms of cellular regulation by GC and GRα. GC bind to GRα, which then dissociates from its chaperone proteins and can regulate target gene expression upon migration to the nucleus. Non-genomic effects of GRα include modulation of apoptotic processes in the mitochondria and direct and indirect (dotted line) regulation of cytoplasmic kinases and NF-κB.

The main mechanisms via which GC drive the transcription and regulation of multiple genes are direct DNA binding, tethering and composite binding (38–40) (Figure 1). In the nucleus, GRα is able to modulate gene transcription through binding to target sequences termed GC-response elements (GREs), largely in the cis-regulatory region of target genes (41). This results in either induction or repression of target gene expression. The binding of the GRα to the GRE can also cause conformational changes in the GR which causes the recruitment of cofactors and coregulators to the site with the ability to modulate and alter the transcriptional rate of many target genes (35, 37). Direct DNA binding of the GRα to GREs results in the induction of gene expression and causes the transcription of multiple genes, including anti-inflammatory genes such as IL-10 and IL-1 receptor antagonist (38, 40) as well as GC-induced leucine zipper (GILZ). GRα can also directly bind to negative GREs (nGRE), which results in suppression of the transcription of several proinflammatory modulators and cytokines including interleukin (IL)-1β (38, 40, 42). Another form of gene repression, termed tethering, is mediated by the ability of GRs to tether to pro-inflammatory transcription factors such as the p65 subunit of nuclear factor kappa B (NF-κB) and activator protein 1 (AP-1), antagonizing their function (35, 43–45). Finally, composite binding encompasses the binding of GRα to a gene locus containing a GRE as well as a binding site for another transcription factor (Figure 1).

Additional factors also influence the ability of GRα to regulate gene transcription such as chromatin structure, epigenetic regulators, proximity to the TATA box, and indirect activation of target genes (38, 41). Next to the genomic actions of GRα, it also affects cellular function through non-genomic mechanisms, including suppression of mitogen activated protein kinases (MAPK) and phosphoinositide 3-kinase (PI3K) signaling pathways and activation of proteins with SRC homology 3 [SH3] domains (46) (Figure 1).

Other factors influencing GC regulation of gene expression include tissue-specific differences in GR expression, and the level of GC dosing (47, 48). Whereas, a single bolus GC dose highly activates GC-mediated biological responses, chronic GC dosing can be associated with GC resistance. Studies in acute adrenalectomized rat models have demonstrated that this resistance is correlated with a down-regulation of GR mRNA and cytosolic receptor density (49, 50). A study by Ayyar et al. found a similar effect of chronic dosing when studying GILZ as a pharmacodynamic marker of GC action in different rat tissues (47). Similar to Ramakrishnan et al. (50) this study also demonstrated drug-induced tolerance during chronic dosing, where GC receptor down-regulation was the main mechanism of regulation (47). Taken together these studies highlight that tissue-specific, and GC-induced, differences in GR levels are important physiological factors to consider in GC responses.

The molecular mechanisms of GRα-mediated effects have been extensively studied, however, much less is known about the GRβ isoform. GRβ results from alternative splicing in exon 9 of NR3C1 and is a truncated isoform that lacks helix 11 and 12 from the ligand-binding domain (51). Since these helices are important for ligand binding, GRβ cannot bind GC and does not directly affect GC-sensitive genes (52). In fact, it is thought that GRβ acts as a dominant negative regulator for GRα. Overexpression of GRβ suppresses GRα-mediated gene transcription, and cells which have higher endogenous levels of GRβ are less responsive to the effects of GC (53–55). How this inhibition occurs is not well-understood, but various mechanisms have been proposed. These include heterodimerization of GRβ with GRα to inactivate GRα, competition for binding at GREs and competition for binding to coactivators such as GRIP1 (53, 56, 57). Furthermore, GRβ also has transcriptional activity independent of GRα and controls its own set of genes (58, 59). Interestingly, GRβ expression is induced by a variety of pro-inflammatory cytokines, such as IL-2, IL-4, IL-17A, IL-17F, IL-23, and TNF-α (60–62). Given their prominent role in inflammatory diseases and the dominant-negative role of GRβ, it is not surprising that high GRβ levels seem to be related to GC resistance in diseases such as asthma, ankylosing spondylitis and SLE (63–65). Furthermore, a polymorphism in *NR3C1* which enhances the stability of GRβ is associated with rheumatoid arthritis (66). Therefore, a better understanding of GRβ biology and how to suppress its function may be an important step toward improving GC-based therapies.

It should be noted that GRα and GRβ are not the only forms of GR. In fact, 27 splice variants of the *NR3C1* gene have currently been identified (67), and hundreds of single nucleotide polymorphisms (SNPs), insertions and deletions which potentially also lead to different variants of the GR proteins have been cataloged (67). Although the physiological role of these variants is currently unknown, they may play a role in individual GC response and therefore warrant further study.

In the context of SLE, GC elicit rapid and potent anti-inflammatory effects upon multiple organs and immune cells. Many of the immune regulatory effects of GC are through direct binding to transcription factors including NF-κB, AP-1, nuclear factor of activated T cells (NFAT) and T-bet (38, 68, 69). This causes a myriad of effects upon immune cells, described in more detail below, and important for the treatment of SLE, the suppression of key mediators of inflammation TNF-α and type I IFNs (9, 14, 70).

### Effect of GC Treatment on Immune Cells Operative in SLE

Thymocytes, particularly double positive CD4^+^CD8^+^TCR^low^ thymocytes, are sensitive to GC-induced apoptosis (71). Cell death can also be induced in mature T cells indirectly by GC-mediated inhibition of IL-2 activation and production (72, 73). GC have been described to affect T cell polarization, shifting the phenotype from Th1 to Th2 (74, 75), however it should be noted that GC affect both T-bet and GATA-3 transcriptional activity, with long-term GC treatment favoring Th2 expansion (75). Further support for polarization toward a Th2 phenotype comes from GC increasing expression of Itk, a Tec kinase able to induce Th2 differentiation through the negative regulation of T-bet (76, 77). GC also increases Treg number and activity, promoting IL-10 producing T cells. This is through several mechanisms: inhibition of activation of T effector cells, GC-mediated Foxp3 induction, and Tregs being more resistant to GC (76).

GC treatment also affects B cells. Firstly, GC induce apoptosis in B cells at all developmental stages (78–80). In addition, GC suppress plasma cell differentiation potentially via down-regulation of Blimp1 and Bcl6 (81, 82). Finally, GC may also directly affect the production of IgG antibodies through inhibition of activation-induced cytidine deaminase (AICDA), an enzyme required for class switch recombination and somatic hypermutation (83). As a result of these GC-induced changes, GC reduce the number of plasma cell precursors and plasma cells and the level of anti-nuclear antibodies demonstrated in the murine MRL/lpr model for SLE (82). Importantly, the number of circulating B cells in human blood is also reduced upon GC treatment (84).

GC also impair dendritic cell (DC) maturation and function (85). DCs treated with GC increase their antigen uptake, decrease their expression of maturation markers (CD80, CD86) and decrease TNF-α, IL-6, and IL-12 production which in turn decreases the induction of T cell responses (85, 86). GC treated DCs have also been described as tolerogenic with the ability to drive T cells toward a Treg phenotype, creating an increase in IL-10 production (87, 88). As a result of these effects, GC enhance the clearance of dead cells and toxins, and increase scavenger function and phagocytosis. GC treatment also decreases the number of pDCs in the peripheral blood, which is important in SLE since they are key IFN-α producers (89). Following GC treatment, levels of IFN-α have been reported to be reduced to levels approximately 25-fold below those seen in untreated healthy donors (89). As type I IFN is implicated in disease severity and activity in SLE, control of pDC number and IFN-α production by GC could be of benefit for the treatment of SLE. Of note, however, pDC IFN production has been reported to be resistant to GC inhibition in SLE, because of TLR-induced NF-κB overcoming GC inhibitory effects (90); it is noteworthy that the IFN signature recognized as associated with SLE is still present in GC-treated patients. Were it to be proven that SLE-related IFN activity was resistant to GC, this would provide a novel target for “assisting” the effects of GC in SLE, for example by reversing factors associated with their inability to suppress IFN in this disease.

Treatment with GC increases the phagocytic ability of macrophages, a finding demonstrated in human macrophages and mouse models (91, 92). This process is assisted by up regulation of mannose receptor (CD206) and scavenger receptors (CD163) on macrophages and enhancement of IL-10 production (93, 94) in response to GC. Significantly for SLE, GC treatment thus also increases the phagocytosis of apoptotic neutrophils by macrophages (95), thus reducing the number of NETs and reducing their pro-inflammatory impact. GC also have direct immune modulating effects on neutrophils, dampening their activation through several mechanisms including anti-apoptotic effects (96), inducing detachment via effects on cell surface CD62L (97) and reducing expression of pro-inflammatory cytokines (98).

**Figure 2:**
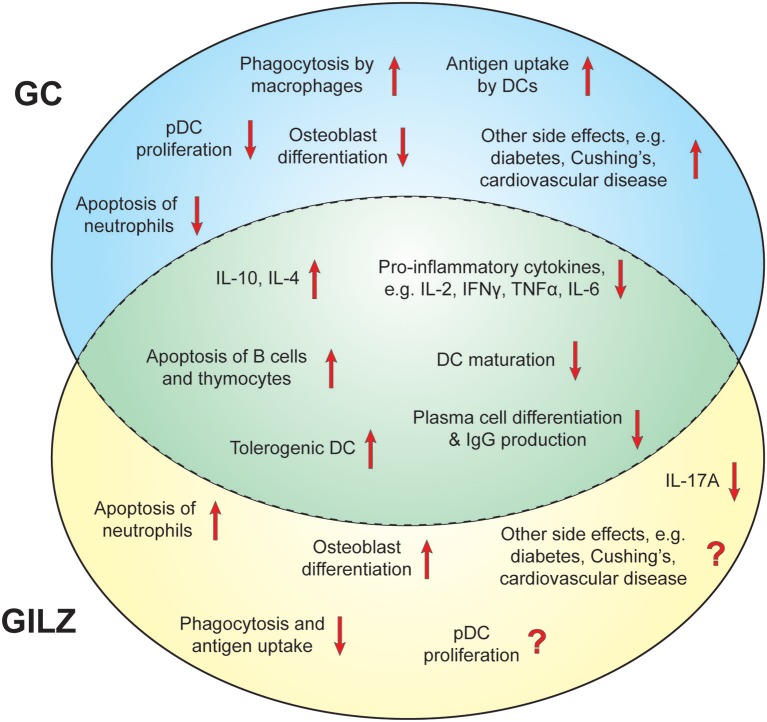
Immunomodulatory effects of GC and GILZ. Overview of the effects of GILZ and GC that contribute to their suppressive capacities and potential side effects in SLE. Unique effects of GC are depicted in the blue circle, unique effects of GILZ in the yellow circle. Shared actions between GC and GILZ are shown in the middle green area.

This wide array of immunomodulation entrained by GC underpins reliance on GC treatment in SLE. Thus, it is imperative that any replacement for GC is able to target multiple immune pathways and provide potent anti-inflammatory immune cell regulation (Figure 2). The ability to achieve these effects without causing the devastating metabolic adverse effects of GC has been described as the “holy grail” of inflammatory pharmacology (99).

## Could GC-induced Leucine Zipper (GILZ) Targeting Be a Replacement for GC Treatment in SLE?

GILZ mRNA expression negatively correlates with SLE disease activity (100) and we have demonstrated that active SLE is associated with lower intracellular GILZ protein levels across multiple leukocyte subsets (11). These findings suggest the possibility that GILZ deficiency contributes to SLE immunopathogenesis, and conversely that GILZ augmentation might be effective in SLE.

GILZ, also known as TSC22 domain family protein 3 (*TSC22D3*) is a 134aa protein in humans. It contains three main regions: an N-terminal domain with a tuberous sclerosis complex (TSC) domain, a leucine zipper, and a proline-rich C-terminal domain. The leucine zipper domain of GILZ largely mediates the homodimerization of GILZ, a requirement for many of its functions (101). The N-terminal and C-terminal domains of GILZ are regions where several protein-protein interactions occur, particularly with transcriptional and signaling molecules. These interactions are critical for the immunosuppressive effects of GILZ.

GILZ has been demonstrated to modulate immune cell activation and promote an anti-inflammatory phenotype (102, 103). This is mediated in part by inhibiting the nuclear translocation and DNA binding of NF-κB, largely through direct binding of the C-terminus of GILZ to the p65 subunit of NF-κB (101, 103). Additionally, GILZ binds to AP-1, a transcription factor consisting of c-Jun and c-Fos with a broad range of functions on immune cell activation (104), GILZ binds to AP-1 through its N-terminal domain and prevents AP-1 from binding to its target DNA (105).

GILZ also interferes with other cellular signaling molecules, such as extracellular signal-regulated kinases (ERK). The ERK pathway is involved in multiple regulatory processes and is key to many immune cell functions included cell differentiation, proliferation, survival, apoptosis, transcription and metabolism (106). GILZ suppresses the ERK pathway via direct binding to Ras via its TSC domain and, depending on the level of Ras activation, via formation of a trimeric complex with Ras and Raf (107, 108). This results in a decrease in activation of downstream targets of Ras and Raf, for example the ERK1/2 and protein kinase B (AKT/PKB) (107), and a subsequent decrease in cell proliferation.

### The Effect of GILZ on Immune Events Involved in SLE Pathogenesis

As noted above, GILZ regulates pivotal transcription factors and cellular pathways involved in immune-inflammatory responses. Notably, many of these transcription factor interactions of GILZ, mimic the transrepressive effects of GC. This raises the potential of GILZ as a replacement for GC. A comparison between the effects of GILZ and GC in animal models of inflammatory diseases has been summarized previously (39).

GILZ has a pro-apoptotic effect on thymocytes similar to GC, as demonstrated by GILZ overexpression systems in mouse models. GILZ overexpression led to a reduction of Bcl-xL expression and increased activation of caspase 3 and 8 (109). This results in a decrease in CD4^+^CD8^+^ thymocytes. However, in T cells, GILZ can exert an anti-apoptotic effect, inhibiting anti-CD3 antibody-induced cell apoptosis through binding to AP-1 (110). This leads to inhibition of FasL expression and inhibition of apoptotic pathways (105, 108).

Similar to GC, GILZ also promotes a Th2 over a Th1 phenotype in T cells. This was demonstrated in activated CD4^+^ T cells from GILZ transgenic mice, which had increased expression of Th2 transcription factors GATA-3 and STAT6 but decreased production of the Th1 transcription factor T-bet (111). Additionally, GILZ induced a Th2 cytokine profile with increased IL-4, IL-5, IL-10, and IL-13 production and a reduction in IFN-γ production. Supporting the role of GILZ in reducing Th1 cytokine production, mouse GILZ-deficient T cells show an increase in IFN-γ production compared to wildtype T cells (112). GILZ also induces the production of Tregs and IL-10, further promoting a regulatory environment and increased production of IL-10 (113).

GILZ regulates cytokine production in T cells and other immune cells largely through inhibition of the transcriptional activity of AP-1, NF-κB, and NFAT transcriptional factors. For example, GILZ binding to NF-κB results in a reduction in IL-2 and IL-2R (103, 114) and regulation of T cell activation (103) and also regulates IL-2 production via binding to AP-1. In human T cells GILZ also inhibits IL-5 production via AP-1 binding, resulting in a negative correlation between expression of IL-5 and GILZ (115). These findings are particularly important for reducing inflammation in a chronic inflammatory disease like SLE, which is characterized by hyperactive T cell responses including T cell-dependent B cell activation.

Next to the classical Th1 and Th2 cells, GILZ also affects Th17 cells. We and others have shown that GILZ inhibits the differentiation of Th17 cells (116, 117). Mechanistically, this is thought to be mediated via direct binding of GILZ to the promoters of Th17 genes including Batf, Stat3, Irf4, and ROR-γt (117). Knockdown of GILZ increases expression of Th17 transcription factors (Rorc, Rbpj, and Batf), cytokines (IL-17A and IL-21) and also reduces Foxp3 expression (117). Furthermore, we have demonstrated, in studies of GILZ-deficient mice, that endogenous GILZ inhibits production of Th17-inducing cytokines IL-1β, IL-23, and IL-6 from bone marrow-derived dendritic cells, further limiting Th17 differentiation (116). Also, using the imiquimod-induced psoriasis model, we have found that GILZ deficiency increases IL-17A, IL-1β, IL-6, and IL-23 in skin lesions. However, these findings may contrast the results of Carceller et al. that demonstrate lesional expression IL-17F, IL-22, and IL-23 increases upon systemic exogenous GILZ overexpression in the same model (116, 118). In human psoriatic lesions, GILZ expression is decreased and correlates negatively with Th17-related pro-inflammatory cytokines IL-23, IL-17A, IL-22, and STAT3 demonstrated in human psoriatic lesions (116).

SLE is characterized by hyperactive B cells and a failure of B cell tolerance to self-antigens. GC have suppressive and cytotoxic effects on B cells (78, 119) and GILZ is able to mimic several of these effects, inhibiting cell proliferation, activation, differentiation, IgG production, and apoptosis (80, 81). We have also demonstrated a reduction in GILZ expression in B cells from both SLE patients and in a lupus prone mouse model (11). Our study also demonstrated that GILZ deficiency results in lupus-like autoimmunity in aged mice, manifesting as excessive B cell responses to T dependent stimulation and the upregulation of genes which promote germinal center B cell phenotype, lupus susceptibility genes and genes for B cell survival and proliferation (11). The consequences of GILZ deficiency *in vivo* in these experiments included spontaneous production of lupus-related autoantibodies including ANA, anti-dsDNA, and anti-Sm, as well as immune complex glomerulonephritis. Additionally, treatment of human B cells with GILZ protein suppressed their responsiveness to T dependent stimuli, providing more evidence that GILZ is a regulator of B cell activity and proof-of-principle that therapeutic supplementation of GILZ could negative regulate B cell activation in SLE.

Deletion of GILZ in mouse models has also been demonstrated to result in an increase in B cell numbers in bone marrow, blood, and lymph nodes (119). This is a corollary of the effect on B cell numbers of GC, which cause a decrease of B cells in several organs and circulation. Thus, GILZ is a key mediator in the regulation of B cell survival. This increase in B cell survival in GILZ-deficient mouse models correlates with an increased NF-κB activity and Bcl-2 expression (119). GILZ regulation of inflammatory immune responses is further demonstrated in a colitis mouse model where GILZ deficient mice had increased IFN-γ production by B cells, increased CD4^+^ T cell activation and enhanced AP-1 activity (120). These mouse models demonstrate proof of principle for the potential therapeutic effect of GILZ in regulating B cell-dependent inflammatory diseases, wherein increased colitis in the setting of GILZ deficiency was reversible via GILZ protein administration (120).

The ability of GILZ to modulate the activation of several signal transduction pathways also affects the maturation of DCs. This is supported in GILZ overexpression models, where GILZ mimicked the inhibitory effects of GC on human DC maturation and activation. For example, GILZ caused a decrease in the expression of DC activation markers (CD80, CD86, and CD83), less IL-12 production and increased IL-10 production (121, 122). GILZ overexpression in DCs can also cause DCs to favor the induction of Tregs over T effector cells (123). Thus, it is suggested that GILZ supports an alternative pathway of activation and differentiation of DCs, leading to a more tolerogenic DC phenotype (122). This has also been demonstrated in mouse studies wherein GILZ affected splenic DC function by inhibiting macropinocytosis and inhibited antigen uptake by CD8a-positive mouse DCs, which had the highest level of GILZ of the splenic DC subsets (124).

Modulation of the NF-κB pathway by GILZ also reduces macrophage activation, as illustrated by the reduced expression of CD80, CD86, TLR 2, and chemokines CCL5 and CCL3 (102). TLR 4 stimulation of GILZ deficient bone marrow derived macrophages results in enhanced NF-κB and AP-1 activity (125). As well as cytokine and chemokine production, GILZ also decreases the phagocytic capacity of macrophages (126). Interestingly, this effect is opposite to the effect of GC, which promote phagocytosis by macrophages. Additionally, GILZ also regulates several neutrophil functions including their activation through inhibition of the MAPK pathway (127), their migration (via annexin A1) (128) and apoptosis (associated with caspases 3, 8, 9) (129, 130).

Interestingly, GILZ is not only regulated by GC, but can also be induced by IL-4, IL-10 and curcumin (102, 131–133). Furthermore, studies in macrophages, *in vitro, ex vivo*, and *in vivo*, have shown that TLR 1/2 and TLR 4 stimulation reduce GILZ mRNA and protein levels (126, 134). Similar findings were reported in T cells, where TCR triggering reduces GILZ expression (103). These data indicate a feedback loop where GILZ is higher in unactivated immune cells and decreases upon their activation.

## Therapeutic Potential and Development of GILZ Delivery

In previous attempts to find safer GC replacement therapies, much research has focussed on selective glucocorticoid receptor agonists and modulators, termed SEGRAMs (135). These compounds were designed to address the hypothesis that GR transactivation was responsible for GC-induced adverse effects, and GR transrepression for anti-inflammatory effects, such that compounds targeting transrepression might be powerfully therapeutic without the side effects of GC. However, it is now known that transactivation effects of GC, such as the induction of GILZ and other GC-induced immune regulators such as DUSP1 (136), are required for the anti-inflammatory effects of GC *in vivo* (137), while adverse effects from GC such as osteoporosis are mediated by both transactivation and transrepression (138).

The potential of GILZ to be the target of a new therapeutic for SLE not only relies upon its immunosuppressive ability but also on a lack of detrimental metabolic effects. Evidence to date is encouraging, although more research is needed. GCs have an inhibitory effect on osteoblast formation, which accounts partially for the rapid bone loss seen in GC-treated patients (139). In contrast, GILZ may exert the opposite effect. Mesenchymal stem cells (MSC) can differentiate into osteoblasts or adipocytes; GILZ expression in MSC increases osteogenic differentiation and inhibits adipocyte formation (140, 141). Furthermore, osteogenic differentiation and development has been shown to be reduced by silencing GILZ (141). The underlying mechanism for this shift in differentiation includes GILZ binding to the tandem repeat of the CCAAT/enhancer binding protein (C/EBP) site in the promotor of peroxisome proliferator-activated receptor gamma-2 (PPARγ2). This decreases PPARγ2 expression, a regulator of adipocyte differentiation (140, 141). Thus, GILZ may play a role in enhancing or stabilizing bone density, rather than inhibiting osteoblast formation and inducing rapid bone loss as seen in GC treated patients. Furthermore, osteoblast-restricted GILZ overexpression resulted in a phenotype characterized by high bone mass, increased bone formation, and increased osteoblast numbers (142). Whereas, the effects of GILZ on osteoblast differentiation are opposite to the effects of GC, a study by Bruscoli et al. indicates that GILZ is required for the anti-myogenic effects of GC on skeletal muscle cells (143). Since both GILZ and GR expression are correlated with protein consumption, this may be mediated by increased protein catabolism that is associated with muscle atrophy (144). In relation to other metabolic adverse effects of GC, such as gluconeogenesis, skin thinning, cataracts and/or cardiovascular side effects, it is still unknown whether GILZ is protective or contributory. Therefore, further studies are essential to determine whether GILZ induces other metabolic adverse effects of GC in order to evaluate its value as a GC replacement therapy.

Studies to date investigating the clinical potential of GILZ-based therapies for autoimmune disease have largely used mouse disease models. Proof of principle studies have demonstrated that local upregulation of GILZ expression through the administration of adeno-associated virus vector system on the day of disease onset inhibits arthritis in the collagen-induced arthritis model (112). Additionally, transgenic mouse models, creating an overexpression of GILZ in T cells, were protective against Th1 mediated colitis (111). *In vitro*, a fusion protein of GILZ with a protein transduction domain (HHpH-GILZ), allowing entry of exogenously applied GILZ to the cell, was able to induce inhibition of Th17 activation and B cell activation (11, 116). Similarly, delivery of GILZ using a transactivator of transcription (TAT)-GILZ fusion protein was able to protect against dinitrobenzene sulfonic acid–induced colitis (145). However, it should be noted that systemic GILZ overexpression may not be beneficial for all disease states. For example, in the murine imiquimod-induced psoriasis model both GILZ deficiency (116) and GILZ transgenic overexpression led to worsening of skin inflammation (118); this is of interest given the clinical finding of glucocorticoid-withdrawal induced flares of psoriasis in humans. Thus, further studies are needed to address the therapeutic utility of GILZ in different disease settings and during established disease states.

Studies utilizing truncated regions of the GILZ protein have also demonstrated therapeutic promise. One study used a peptide targeting the C-terminus region of GILZ 115-137aa from the mouse GILZ sequence. This region binds to the p65 subunit of NF-κB, inhibiting NF-κB translocation to the nucleus and DNA binding (146). This peptide was demonstrated to have therapeutic potential in experimental autoimmune encephalitis (EAE), where it decreased T cell proliferation, decreased IL-12, IFN-γ, and IL-17 production and increased IL-10 production (146). Furthermore, the GILZ peptide decreased T-bet mRNA and increased GATA-3 mRNA levels creating a Th2 T cell phenotype. A single dose of GILZ peptide on day of disease induction was protective against the development of EAE in mice (146). Another study, utilizing a similar region of GILZ 98-134aa of the human GILZ sequence, demonstrated the anti-inflammatory activity GILZ (147). Here GILZ administration similarly suppressed the nuclear translocation of NF-κB, inhibited cytokine production and inhibited the gliosis of Muller cells (147).

Although these studies utilizing the C-terminus of GILZ are promising for use of a GILZ-based therapy in autoimmune diseases, it should be noted that the N-terminal domain of GILZ is also important for inhibition of transcription factors and signaling pathways. Therefore, all or several regions of GILZ may be required for the potent regulation of multiple inflammatory immune responses, an essential requirement of a new SLE therapy. This implies that strategies targeting the endogenous expression of natural GILZ, or its degradation, may hold greater promise. A greater understanding of the mechanisms of regulation of GILZ and its gene targets is critical to advance this field. This knowledge is also important for a potential gene therapy approach to deliver GILZ, either via expressing peptides or the entire protein. An additional method which may hold promise as a future therapeutic mechanism could be to induce GILZ expression using small molecules. For example, two SEGRAM compounds under investigation, RU24858 and ORG 214007-0, induce GILZ (135). We consider that methods to induce GILZ expression that do not utilize the GR could avoid GR-dependent metabolic effects. Additionally, a GC replacement therapy which delivers or targets GILZ could alleviate the effects of GR down-regulation by chronic GC dosing. Thus, evaluation of multiple pathways is required to lead to the development of a therapy to therapeutically induce GILZ. As GILZ is also a *bona fide* transcription factor (117), studies cataloging in full the gene targets of GILZ are also required in order to understand the targets for GILZ mimics, both for comprehending the potential for potent immune modifying effects as well as potential adverse effects.

## Summary

SLE is a complex chronic autoimmune disease characterized by heterogeneous clinical features as a consequence of a failure of multiple immune checkpoints. This leads to hyperactive B and T cell responses, the production of autoantibodies and formation of immune complexes, activation of innate immunity, and consequently inflammation, organ damage, morbidity and mortality. Currently there is no cure for SLE and mainstay treatment with GC causes debilitating adverse effects. GILZ represents a novel target for the induction of a potent anti-inflammatory and immune suppressive response targeting multiple signaling pathways and immune cells. Proof of principle studies have demonstrated GILZ to have significant therapeutic effects in animal models of autoimmune disease. The immunosuppressive effects of GILZ are broadly similar to those of GC and to date, other than myogenic effects, there is no evidence of adverse metabolic effects of GILZ. Further research is required, to determine whether a GILZ based therapy would have metabolic effects, and into the molecular mechanisms for the induction and targeting of GILZ. The idea of a new therapy which enhances GILZ expression, and/or targets the same molecular pathways as GILZ, is a very attractive one with the potential to provide a critically-needed replacement for GC therapy in SLE.

## Author Contributions

JF and EM designed the review. JF and WD wrote the review. All authors critically revised the manuscript and approved it for publication.

### Conflict of Interest Statement

The authors declare that the research was conducted in the absence of any commercial or financial relationships that could be construed as a potential conflict of interest.
